# The expression characteristic and prognostic role of Siglec‐15 in lung adenocarcinoma

**DOI:** 10.1111/crj.13772

**Published:** 2024-05-09

**Authors:** Haijun Sun, Qilong Du, Yuyu Xu, Cheng Rao, Li Xu, Junrong Yang, Yuan Mao, Lin Wang

**Affiliations:** ^1^ Department of Thoracic Surgery The First People's Hospital of Lianyungang Lianyungang China; ^2^ The First Affiliated Hospital of Kangda College of Nanjing Medical University/The First People's Hospital of Lianyungang Lianyungang China; ^3^ The Affiliated Lianyungang Hospital of Xuzhou Medical University/The First People's Hospital of Lianyungang Lianyungang China; ^4^ Lianyungang Clinical College of Nanjing Medical University/The First People's Hospital of Lianyungang Lianyungang China; ^5^ Department of Oncology The Fourth Affiliated Hospital of Nanjing Medical University Nanjing China; ^6^ Department of Central Laboratory The Fourth Affiliated Hospital of Nanjing Medical University Nanjing China; ^7^ Department of Pathology, Jiangsu Cancer Hospital Affiliated Cancer Hospital of Nanjing Medical University Nanjing China; ^8^ Department of Pathology The Fourth Affiliated Hospital of Nanjing Medical University Nanjing China; ^9^ Department of Hematology and Oncology Geriatric Hospital of Nanjing Medical University, Jiangsu Province Geriatric Hospital Nanjing China

**Keywords:** immunotherapy, LUAD, prognosis, Siglec‐15

## Abstract

Sialic acid‐binding immunoglobulin‐like lectin‐15 (Siglec‐15) has been identified as an immune suppressor and a promising candidate for immunotherapy of cancer management. However, the association between Siglec‐15 expression and clinicopathological features of lung adenocarcinoma (LUAD), especially the prognostic role, is not fully elucidated. In this present study, a serial of bioinformatics analyses in both tissue and cell levels were conducted to provide an overview of Siglec‐15 expression. Real‐time quantitative PCR (qPCR) test, western blotting assay, and immunohistochemistry (IHC) analyses were conducted to evaluate the expression of Siglec‐15 in LUAD. Survival analysis and Kaplan–Meier curve were employed to describe the prognostic parameters of LUAD. The results of bioinformatics analyses demonstrated the up‐regulation of Siglec‐15 expression in LUAD. The data of qPCR, western blotting, and IHC analyses further proved that the expression of Siglec‐15 in LUAD tissues was significantly increased than that in noncancerous tissues. Moreover, the expression level of Siglec‐15 protein in LUAD was substantially associated with TNM stage. LUAD cases with up‐regulated Siglec‐15 expression, positive N status, and advance TNM stage suffered a critical unfavorable prognosis. In conclusion, Siglec‐15 could be identified as a novel prognostic biomarker in LUAD and targeting Siglec‐15 may provide a promising strategy for LUAD immunotherapy.

## Graphical abstract

The expression of sialic acid‐bound immunoglobulin lectin 15 (Siglec‐15) was significantly increased than that in noncancerous tissues. The expression level of Siglec‐15 protein in LUAD was statistically associated with TNM stage. Siglec‐15 could be identified as a novel prognostic biomarker in LUAD, and targeting Siglec‐15 may provide a promising strategy for LUAD immunotherapy.

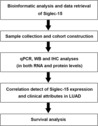

## 1 INTRODUCTION

Lung cancer (LC) is one of the most common cancer and remains the leading cause of cancer‐related death worldwide.^1^ In China, the current situation of LC is severely frustrating because the mortality of LC has been increasing by more than 400% over the past three decades.^2^ According to the pathological features, the two major types of LC are small cell lung carcinoma (SCLC) and non‐small cell lung carcinoma (NSCLC). Lung adenocarcinoma (LUAD) is the most common subtype of NSCLC and is characterized by high invasiveness, noticeable metastasis, and poor prognosis.^3^ For now, molecular targeted therapies have demonstrated significant effectiveness in elongating overall survival (OS) in LUAD patients with positive driver gene mutations.^4^ Immunotherapy also showed marvelous outcome for LUAD patients by utilizing immune checkpoint inhibitors (ICIs), including anti(a)‐PD‐1, aPD‐L1, and aCTLA‐4 antibodies.^5^, ^6^ However, only a part of LUAD patients could respond to targeted therapy and immunotherapy, and drug‐resistance was eventually inevitable.^7^, ^8^ Hence, the screen and identification of new biomarkers, especially novel checkpoints with clinical potential, are of great importance and urgent need.

Sialic acid‐binding immunoglobulin‐like lectins (Siglecs) are a family of sialic acid immunoglobulin receptors that play important roles in recognizing sialylated glycans and in regulating immune homeostasis.^9^ Recently, an increasing number of Siglec members have been found to play a crucial role in tumor immunosuppression.^10^ Siglec‐15, also known as CD33L3, is a special family member of Siglecs that presents one IgV and one IgC2 domain, demonstrating distinct similarity with B7 family molecules.^11^ Siglec‐15 expression is primarily observed in human dendritic cells and macrophages.^12^ In tumor microenvironment (TME), tumor cells with high Siglec‐15 expression often demonstrate highly malignant features and behaviors.^13^ Lately, Siglec‐15 has been reported to act as an novel immune checkpoint molecule and could be identified as a suitable candidate for cancer immunotherapy.^14^ A number of studies describe that Siglec‐15 expression is reciprocally particular to PD‐L1 in many solid tumors, including NSCLC.^15^ A research recently stated that Siglec‐15 works individually of the PD‐1/PD‐L1 pathway in TME, implying that suspending Siglec‐15 action may deliver an alternative immune therapy for those patients who failed to respond to initial PD‐1/PD‐L1 therapy.^16^ However, whether Siglec‐15 also works oncogenically in LUAD and whether Siglec‐15 could be identified as a valuable biomarker correlating important clinical parameters of LUAD, relative studies are rare.

In this study, a number of bioinformatic databases were first consulted. Then we collected LUAD tissue samples to examine the expression of Siglec‐15 expression in both mRNA and protein levels. The relationship between Siglec‐15 expression and clinicopathologic attributes was further explored. The prognostic role of Siglec‐15 in LUAD was finally evaluated.

## 2 MATERIALS AND METHODS

### 2.1 Bioinformatic analysis and data retrieval

The Human Protein Atlas (HPA) database was examined to explore the skeletal and detailed expression characteristics of Siglec‐15 (http://www.proteinatlas.org/). Gene Expression Profiling Interactive Analysis (GEPIA) database was searched to investigate the expression status of Siglec‐15 in various solid tumors (http://gepia.cancer-pku.cn/). TCGA database was further consulted to confirm the mRNA express of Siglec‐15 (https://cancergenome.nih.gov). Kmplot database was employed to detect the prognostic function of Siglec‐15 (http://kmplot.com/analysis/).

### 2.2 Tissue samples

Sixteen fresh LUAD tissue samples and corresponding noncancerous tissue samples were collected from the Department of Thoracic Surgery, The First People's Hospital of Lianyungang from Jan 2020 to Dec 2022. A total of 93 formalin‐fixed, paraffin‐embedded LUAD samples and 89 corresponding noncancerous samples were collected from Outdo Biotech Co., Ltd (Shanghai, China). Important clinicopathological data of LUAD cases were provided from the raw data along with the TMA product. Clinical staging was defined based on the American Joint Committee on Cancer/International Union Against Cancer TNM staging system. Written informed consent was also collected from LC patients enrolled in the present research. Ethical and research protocols were approved by the Human Research Ethics Committee of The Fourth Affiliated Hospital of Nanjing Medical University.

### 2.3 One‐step qPCR test and western blotting analysis

For qPCR test, total RNA was extracted from 16 cases of the frozen LUAD tissue samples using the Trizol reagent following the manufacturer's protocols. The detailed experiment of RNA extraction and qPCR analysis were performed as previously described.^17^ For western blotting analysis, total protein was separated and collected from three LUAD tissue samples and transferred onto nitrocellulose membrane. The membranes were first incubated with the polyclonal Siglec‐15 antibody (NBP2‐41162, Novus Biologicals, USA) and then were detected by ECL kit. The detailed protocol was described previously.^18^

### 2.4 Immunohistochemistry (IHC) analysis

IHC analysis was performed as previously described.^17^ Tissue sections were incubated with polyclonal rabbit anti‐Siglec‐15 antibody (abcam, ab198684, 1:150) in TBS. Siglec‐15 immunostaining score was examined by two autonomous pathologists on the basis of intensity and percentage of positive staining cells. The detailed protocol was described in our previous studies.^19^, ^20^ Briefly, the degree of Siglec‐15 staining was defined as follows: Samples with a final score <4 were recognized as low expression while those with a final score ≥4 were determined as high expression. Samples with a final score = 0 were classified as negative expression.

### 2.5 Statistical analysis

All values were showed as the mean ± standard error. The relationships between Siglec‐15 expression and important clinical parameters were analyzed by chi‐square tests. Survival rate was explored by Kaplan–Meier method. Univariate and multivariate analyses were performed by utilizing Cox's proportional hazards regression models to identify and validate prognostic factors. *P* < 0.05 was considered to indicate a statistically significant difference. All statistical analyses were performed by using STATA 18.0 (Stata Corporation, College Station, TX, USA).

## 3 RESULTS

### 3.1 Bioinformatic summary of Siglec‐15 expression in human tissues

**FIGURE 1:**
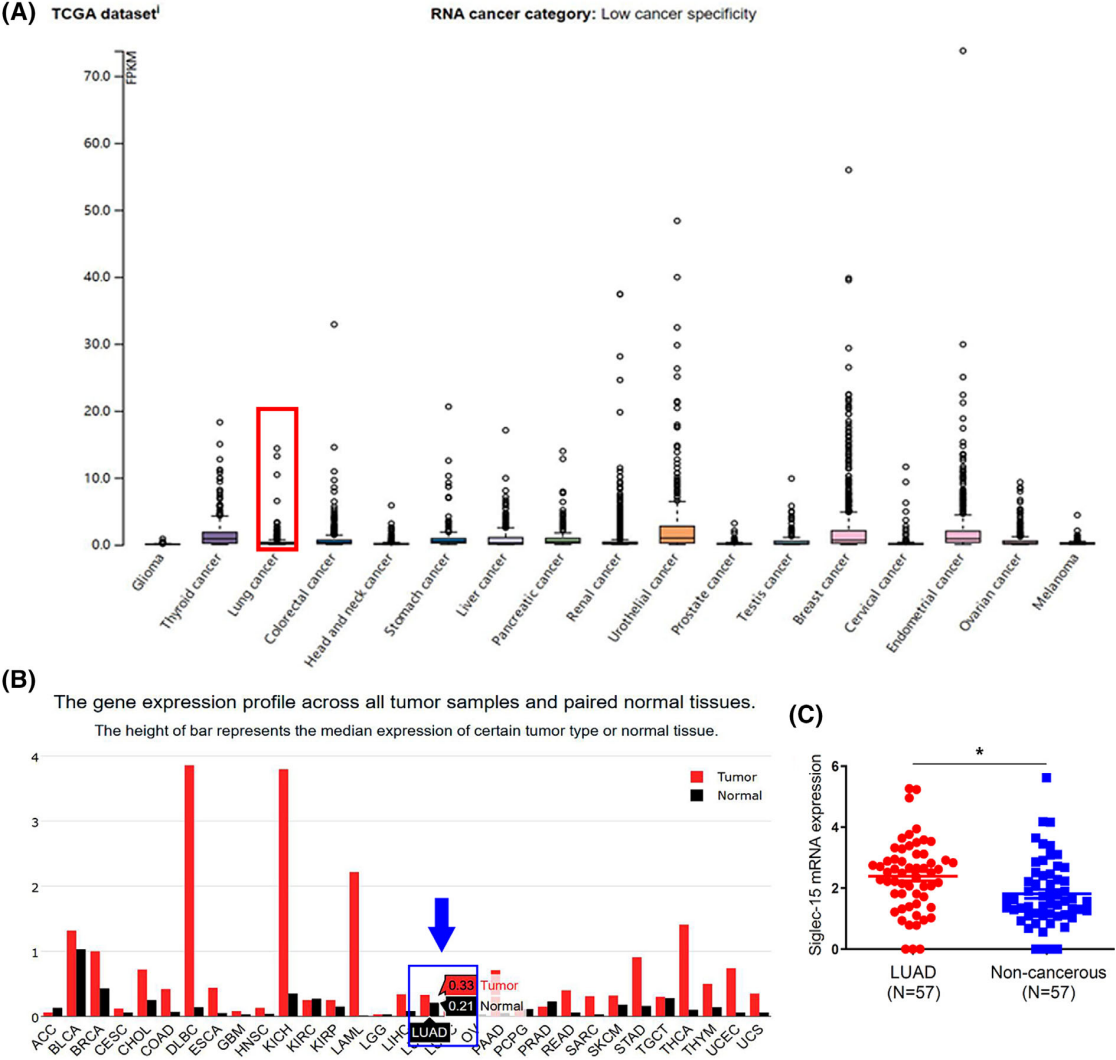
(A) The Human Protein Atlas (HPA) database (http://www.proteinatlas.org/) showed the overview of Siglec‐15 expression in 17 human cancers based on TCGA database and the data indicated the low cancer specificity of Siglec‐15 expression. (B) Gene Expression Profiling Interactive Analysis (GEPIA) database (http://gepia.cancer‐pku.cn/) demonstrated the outline of Siglec‐15 expression in 31 human cancers. Blue arrow and frame marked the Siglec‐15 expression in LC tissue (0.33) and normal tissue (0.21). (C) TCGA database (https://cancergenome.nih.gov) illustrated that the RNA expression of Siglec‐15 in 57 paired LUAD tissues (2.39 ± 1.16) was significantly higher than that in corresponding noncancerous tissues (1.81 ± 1.11) (*P* = 0.0078, *t* = 2.711, *df* = 112).

The HPA database provided the overview of Siglec‐15 expression in 17 human cancers based on TCGA database, and the data indicated that Siglec‐15 expression was low cancer specificity (Figure 1A and Figure S1). The GEPIA database described the expression status of Siglec‐15 in 31 human cancer samples compared with that of in noncancerous samples (Figure 1B). The data of TCGA database further confirmed that the RNA expression of Siglec‐15 in LUAD tissues was significantly higher than that in corresponding noncancerous tissues (Figure 1C).

### 3.2 Bioinformatic outline of Siglec‐15 expression in cancer cells

**FIGURE 2:**
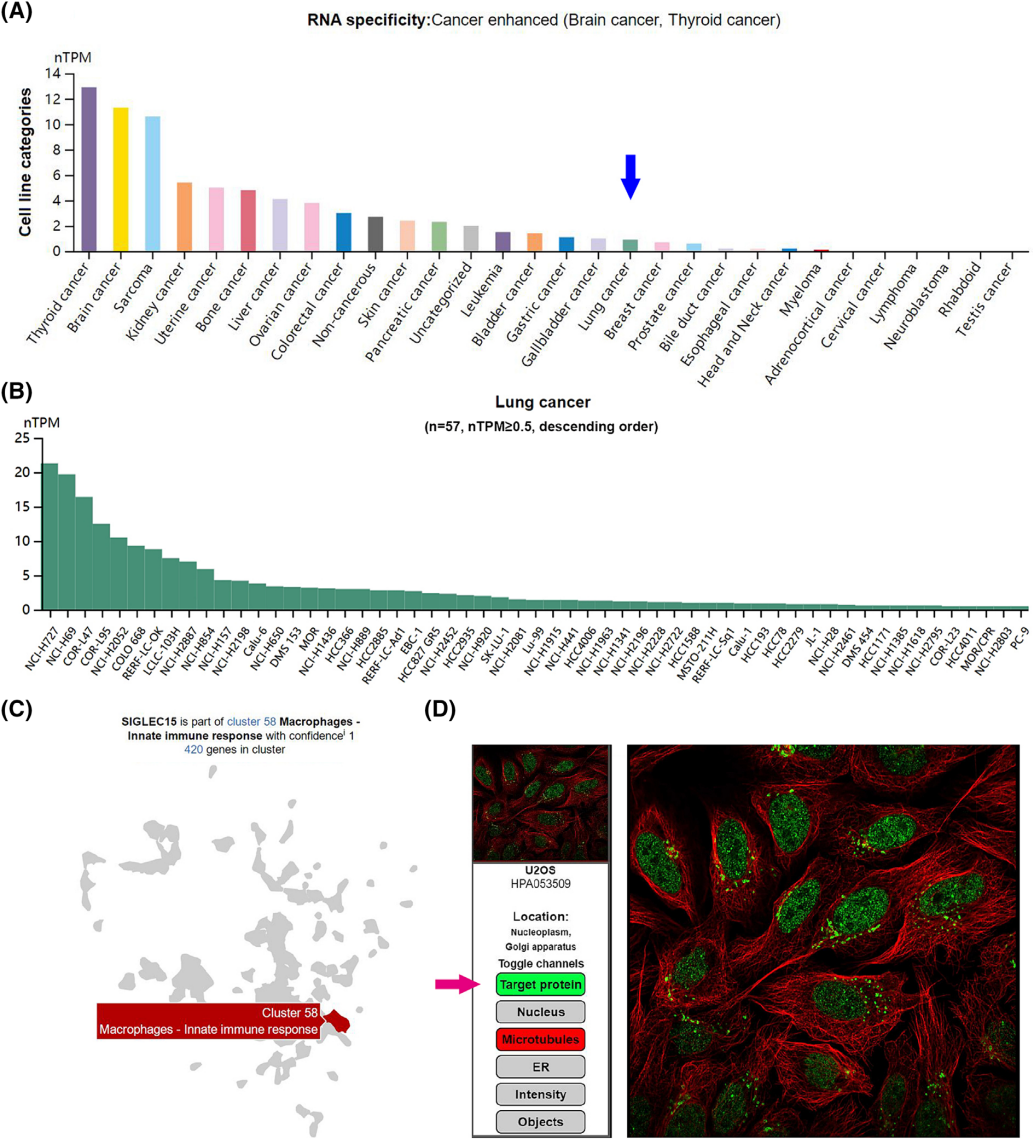
(A) HPA database introduced the Siglec‐15 expression in various cancer cell lines. Particularly, Siglec‐15 expression in brain cancer and thyroid cancer was significantly up‐regulated. Blue arrow marked the Siglec‐15 expression in lung cancer cell line. (B) Summarization of Siglec‐15 expression in 57 lung cancer cell lines (nTPM ≥ 0.5). (C) Single‐cell sequencing data demonstrated that Siglec‐15 expression was dominantly observed in macrophages. (D) The typical location of Siglec‐15 protein was in nucleoplasm. Target protein was marked by green fluorescence and highlighted by a pink arrow.

HPA database introduced the Siglec‐15 expression in various cancer cell lines. Particularly, Siglec‐15 expression in brain cancer and thyroid cancer was significantly up‐regulated (Figure 2A). Then the detailed information of Siglec‐15 expression in LC cell lines was demonstrated in Figure 2B. For single‐cell sequencing level, Siglec‐15 expression was dominantly witnessed in macrophages (Figure 2C). Figure 2D showed the typical location of Siglec‐15 protein in cell was nucleoplasm.

### 3.3 Bioinformatic information of the prognostic roles of Siglec‐15

**FIGURE 3:**
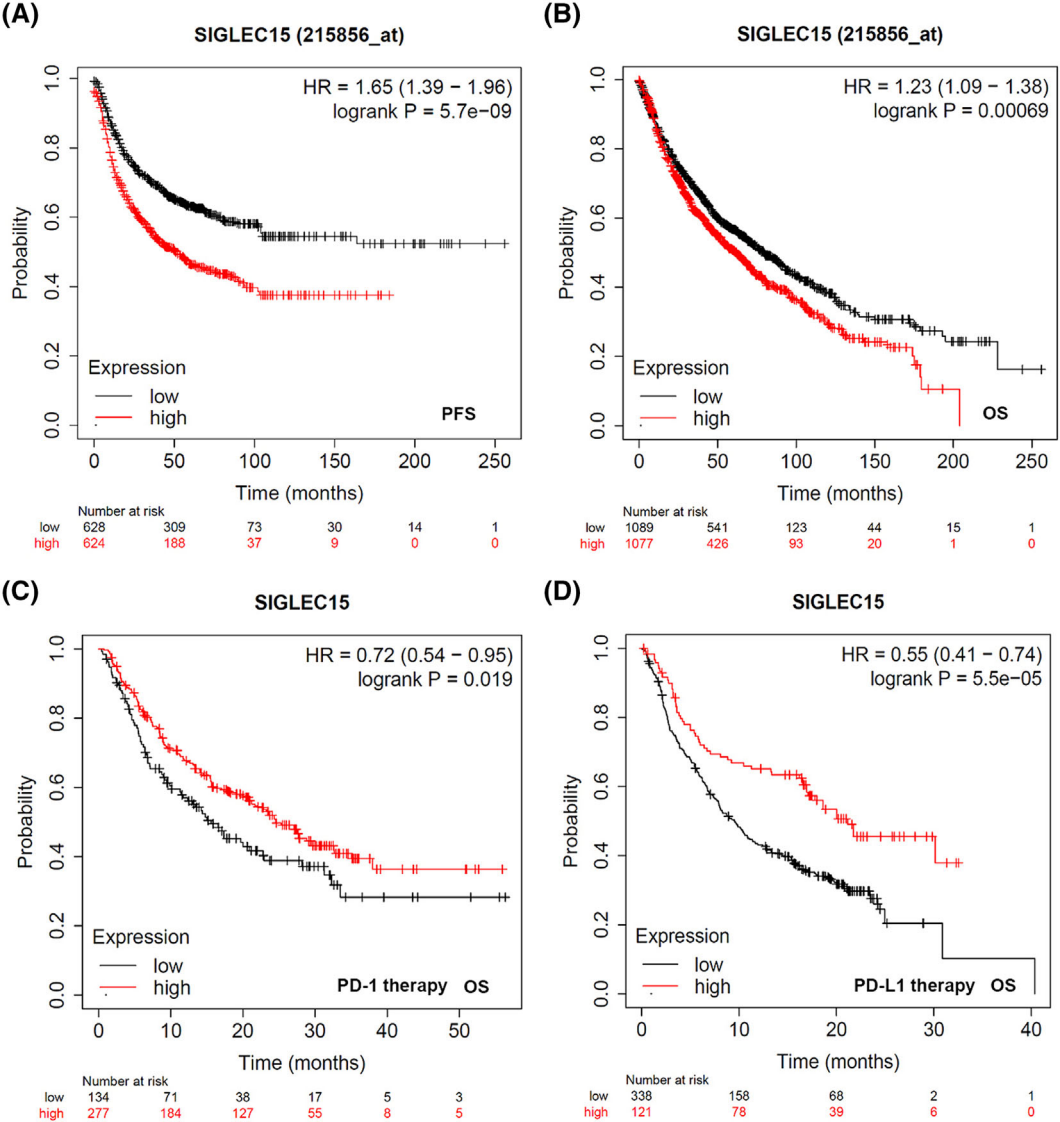
Kmplot database (http://kmplot.com/analysis/) was employed to describe the prognostic characteristics of Siglec‐15. (A,B) Elevated Siglec‐15 expression indicated poor prognosis for both progression free survival (PFS, *P* = 5.7 × 10^−9^) and overall survival (OS, *P* = 0.00069). (C,D) High Siglec‐15 expression implied favorable overall survival (OS) when treated with PD‐1 (*P* = 0.019) or PD‐L1 (*P* = 5.5 × 10^−5^).

Kmplot database was investigated to examine the prognostic characteristics of Siglec‐15. Figure S2 exhibited that high Siglec‐15 expression suggests poor prognosis in Pan‐cancer circumstance (*P* = 0.0299). Moreover, for LC, elevated Siglec‐15 expression also indicated poor prognosis for both progression free survival (PFS, *P* = 5.7 × 10^−9^) and overall survival (OS, *P* = 0.00069) (Figure 3A,B). In addition, Figure 3C,D showed that high Siglec‐15 expression implied favorable overall survival (OS) when treated with PD‐1 (*P* = 0.019) or PD‐L1 (*P* = 5.5 × 10^−5^).

### 3.4 Siglec‐15 expression was up‐regulated in LUAD

**FIGURE 4:**
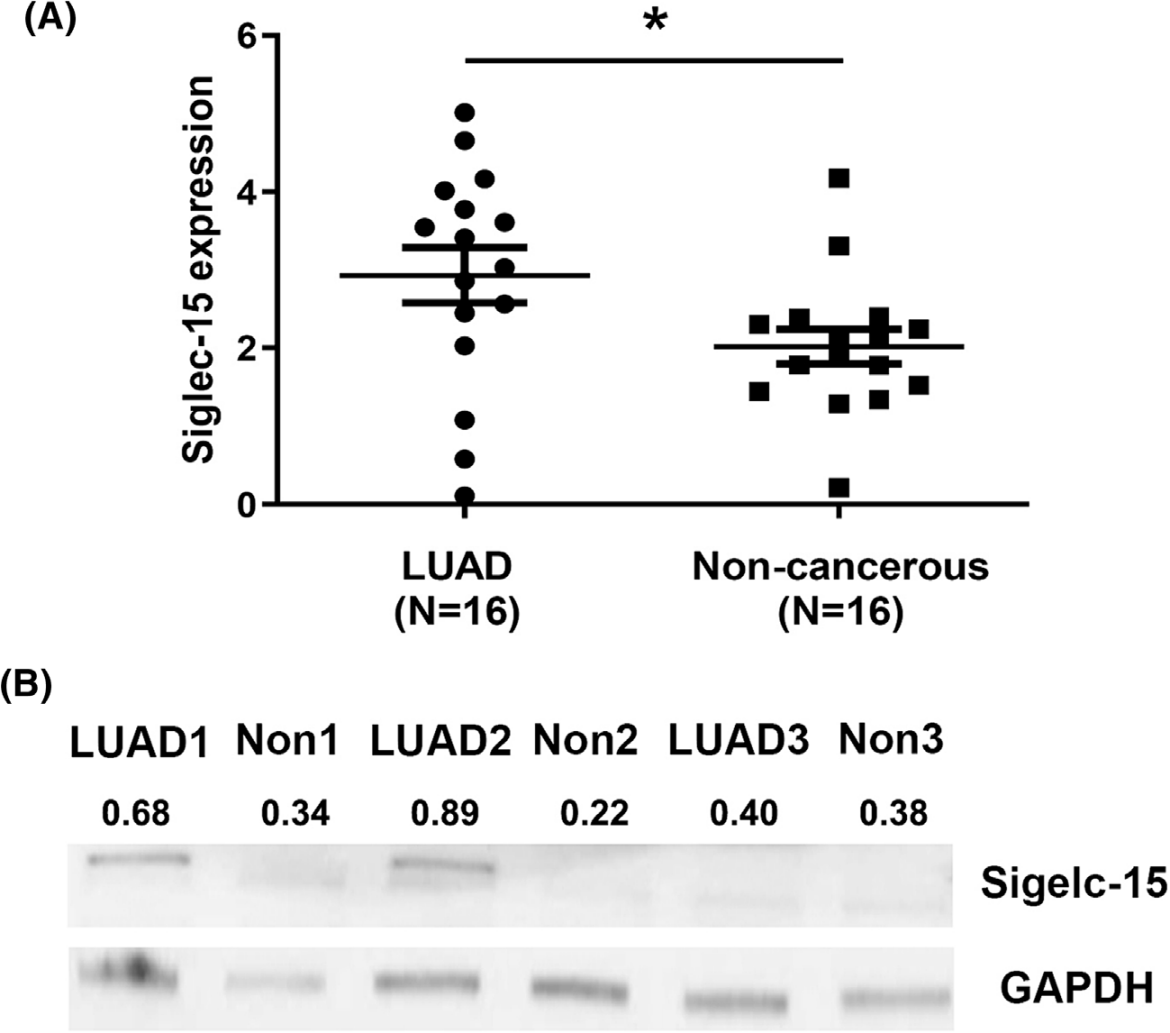
(A) The result of one‐step quantitative real‐time polymerase chain reaction (qPCR) test showed that the expression of Siglec‐15 mRNA in LUAD tissues (2.928 ± 1.41) was significantly higher than that in matched noncancerous tissues (2.019 ± 0.88), when normalized to GAPDH (*P* = 0.0369, *t* = 2.184, *df* = 30). (B) The data of Western blotting analysis validated the results of the previous qPCR test. In three LUAD cases, the Siglec‐15 protein expression was tremendously higher in cancer tissues than that in noncancerous tissues. LUAD: lung adenocarcinoma tissue samples; Non: noncancerous tissue samples.

Sixteen LUAD tissue samples were collected for qPCR test. When normalized to GAPDH, the means of Siglec‐15 mRNA in LUAD and corresponding noncancerous tissues were 2.928 ± 1.41 and 2.019 ± 0.88, respectively (*P* = 0.0369). The Siglec‐15 expression averaged 1.45‐fold higher in the LUAD tissues than in noncancerous tissues (Figure 4A). Three LUAD cases were then subject to western blotting analysis. The results demonstrated that Siglec‐15 protein expression in LUAD tissues was significantly elevated compared with that in noncancerous tissues, which confirmed the data obtained from qPCR test (Figure 4B).

### 3.5 Detection of Siglec‐15 protein expression by IHC analysis

**FIGURE 5:**
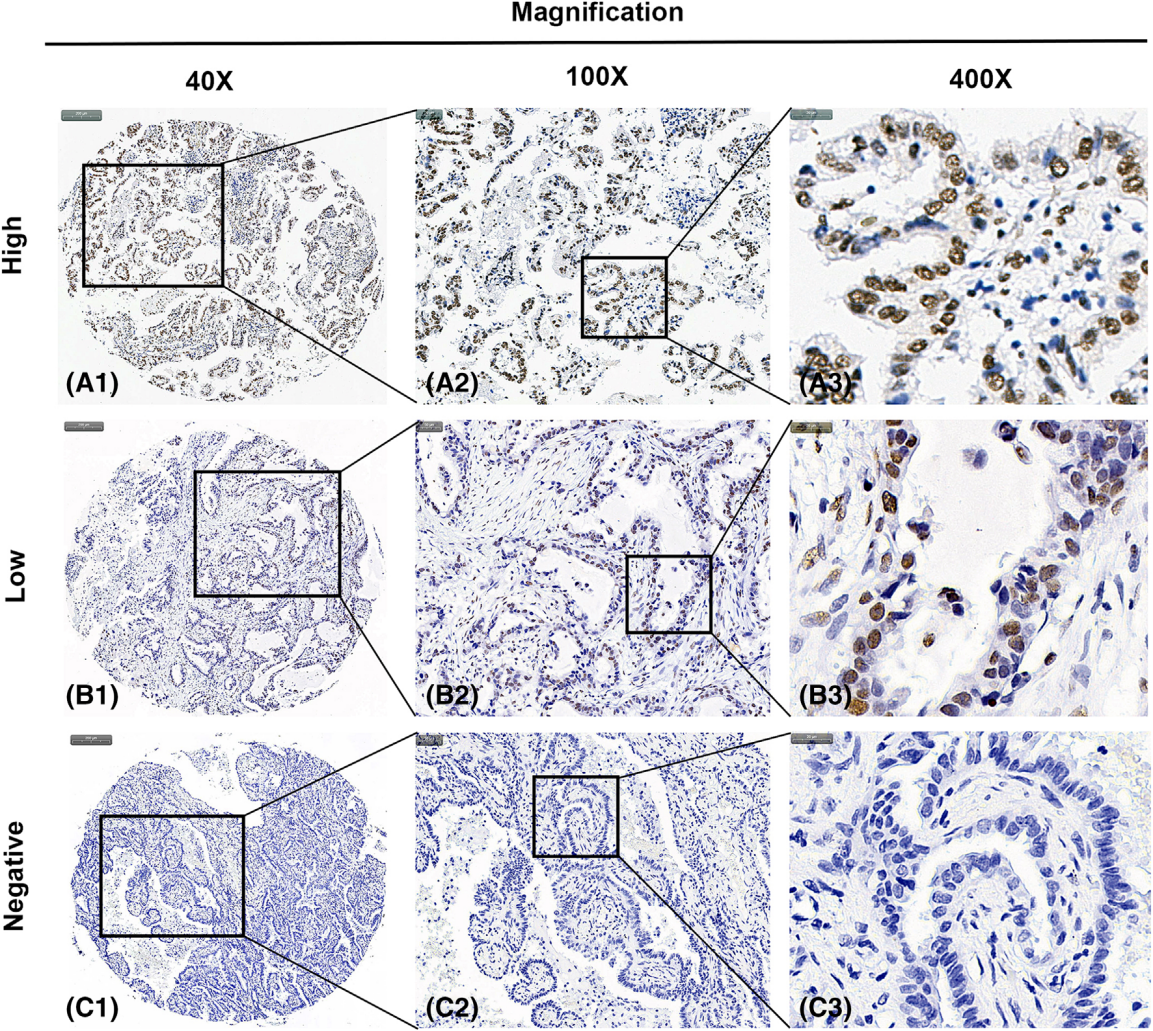
Representative images of Siglec‐15 protein expression in LUAD samples with tissue microarray (TMA). IHC staining of Siglec‐15 protein was shown as brown. A1, A2, and A3. High immunohistochemical (IHC) staining of Siglec‐15 protein in LUAD samples. B1, B2, and B3. Low IHC staining of Siglec‐15 protein in LUAD samples. C1, C2, and C3. Negative IHC staining of Siglec‐15 protein in LUAD samples. ×40 in A1, B1, and C1; ×100 in A2, B2, and C2; ×400 in A3, B3, and C3.

**FIGURE 6:**
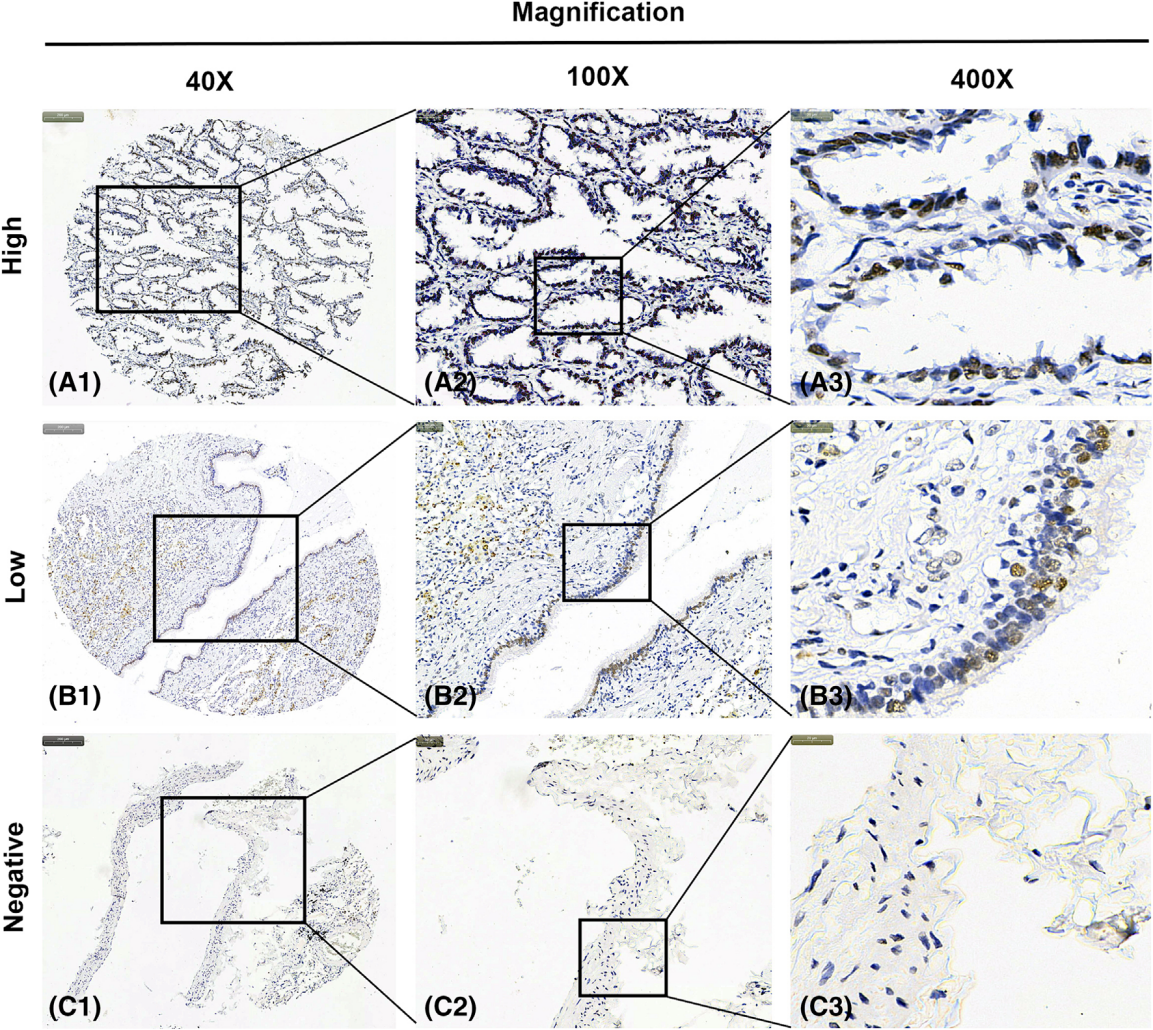
Representative images of Siglec‐15 protein expression in noncancerous samples with tissue microarray (TMA). Immunohistochemistry (IHC) staining of Siglec‐15 protein was shown as brown. A1, A2, and A3. High immunohistochemical (IHC) staining of Siglec‐15 protein in noncancerous samples. B1, B2, and B3. Low IHC staining of Siglec‐15 protein in noncancerous samples. C1, C2, and C3. Negative IHC staining of Siglec‐15 protein in noncancerous samples. ×40 in A1, B1, and C1; ×100 in A2, B2, and C2; ×400 in A3, B3, and C3.

IHC analysis was executed to examine the protein expression of Siglec‐15 in LUAD. In this cohort, high Siglec‐15 expression was detected in 30 (32.3%) of 93 LUAD tissues compared with 14 (15.7%) of the matched noncancerous tissues. The result showed statistical significance (*P* < 0.05) and were in accordance with the data that high Siglec‐15 expression was more frequently observed in LUAD tissues using qPCR and western blotting analyses. Positive staining was largely witnessed in the nuclei of LUAD cells. Representative pictures for Siglec‐15 staining in LUAD tissues and noncancerous tissues are shown in Figures 5 and 6, respectively. Specifically, Figure 5 demonstrated the Siglec‐15 expression in LUAD tissue samples while Figure 6 displayed the Siglec‐15 expression in noncancerous tissue samples. High Siglec‐15 expression was substantially associated with TNM stage (*P* = 0.019) (Table 1).

**TABLE 1:** Correlation of high Siglec‐15 protein expression with clinicopathological characteristics in 93 LUAD.

Groups	No.	Siglec‐15		*χ* ^2^	*P* value
		+	%		
Gender					
Male	50	17	34.0	0.15	0.698
Female	43	13	30.2		
Age					
≥60 years	57	17	29.8	0.40	0.528
<60 years	36	13	36.1		
Tumor diameter					
≥3 cm	60	20	33.3	0.09	0.765
<3 cm	33	10	30.3		
Pathological grade					
Grade I–II	66	18	27.3	2.59	0.108
Grade III	27	12	44.4		
T status					
T1–T2	70	20	28.6	1.76	0.185
T3–T4	23	10	43.5		
N status					
Positive	38	15	39.5	2.15	0.143
Negative	52	13	25.0		
Insufficient data	3				
M status					
Positive	1	0	0.0	0.49	0.484
Negative	91	30	34.4		
Insufficient data	1				
TNM stage					
Stage I–II	45	9	20.0	5.54	0.019*
Stage III–IV	44	19	43.2		
Insufficient data	4				
Prognosis					
Live	22	8	33.3	0.22	0.637
Dead	71	22	31.9		

* *P* < 0.05.

### 3.6 Survival analysis

**FIGURE 7:**
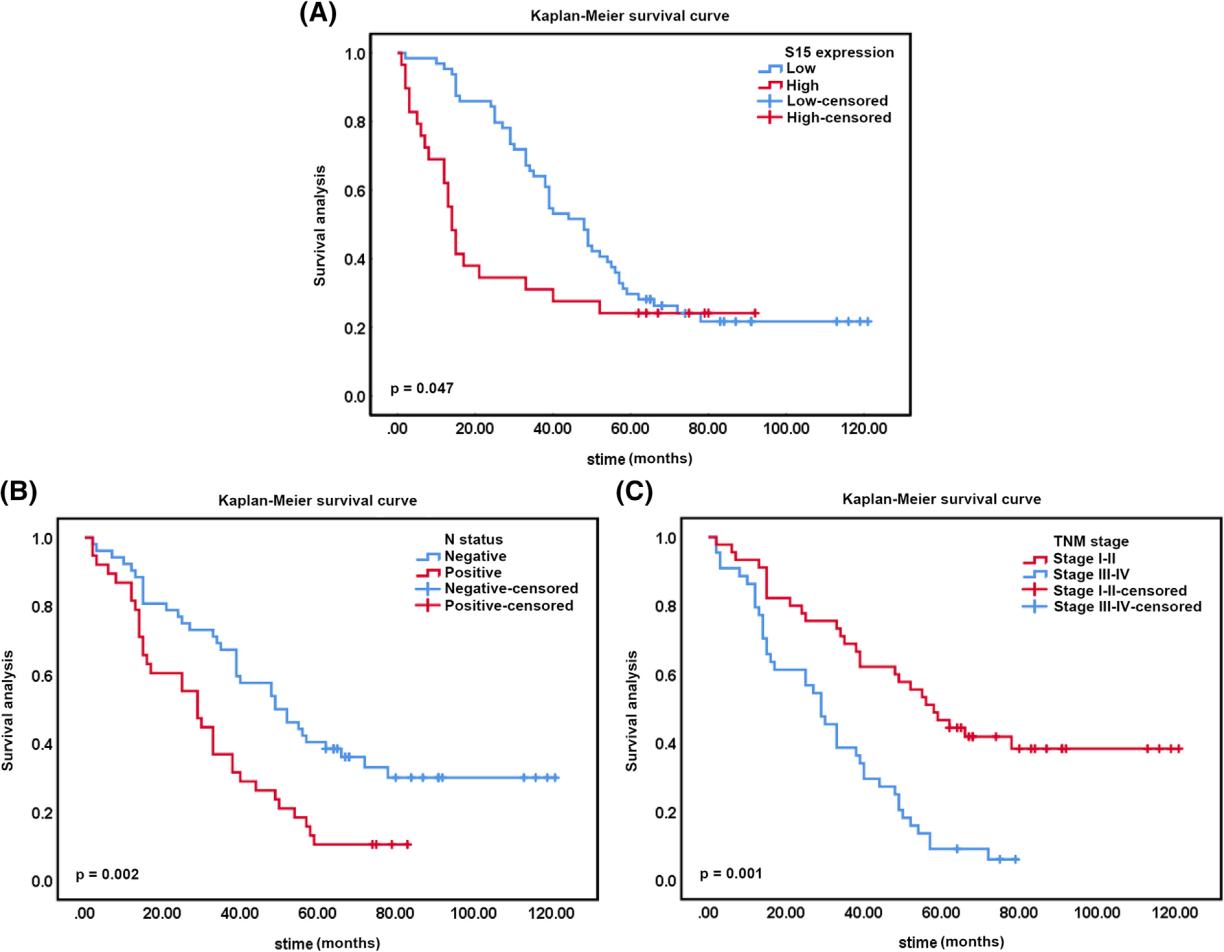
Survival analysis of 93 LUAD patients by Kaplan–Meier method. (A) Overall survival rate in patients with high Siglec‐15 expression (red line) was significantly lower than that in patients with low Siglec‐15 expression (blue line). (B) Overall survival rate in patients with positive N status (red line) was significantly lower than that in patients with negative N status (blue line). (C) Overall survival rate in patients with advance TNM stage (stage III–IV, blue line) was significantly lower than that in patients with early TNM stage (stage I–II, red line).

Univariate analysis was conducted to screen the prognostic element affecting LUAD outcome in this cohort. The results showed that four factors including Siglec‐15 expression (*P* = 0.047), N status (*P* = 0.002), and TNM stage (*P* = 0.001) demonstrated a significant association with the overall survival of LUAD patients. Multivariate analysis confirmed that TNM stage (*P* = 0.036) could be considered as an independent prognostic factors in this LUAD cohort (Table 2). Kaplan–Meier survival curves showed that LUAD patients with high Siglec‐15 expression, positive N status, and advance TNM stage encountered a remarkably unfavorable overall survival time (Figure 7).

**TABLE 2:** Univariate and multivariate analysis of prognostic factors for overall survival in 93 LUAD patients.

	Univariate analysis			Multivariate analysis		
	HR	*P* value	95% CI	HR	*P* value	95% CI
Siglec‐15 expression						
High vs. low	1.67	0.047*	1.01–2.77	1.16	0.592	0.67–2.01
Gender						
Male vs. female	1.38	0.183	0.86–2.20			
Age						
≥60 years vs. <60 years	0.96	0.861	0.59–1.55			
Tumor diameter						
≥3 cm vs. <3 cm	1.54	0.090	0.93–2.55			
Pathological grade						
Grade I–II vs. Grade III	0.88	0.633	0.53–1.47			
T status						
T1–T2 vs. T3–T4	0.70	0.196	0.42–1.20			
N status						
Positive vs. negative	2.11	0.002*	1.31–3.41	1.51	0.191	0.81–2.82
M status						
Positive vs. negative	1.09	0.930	0.15–7.91			
TNM stage						
Stage I–II vs. Stage III–IV	0.36	0.001*	0.21–0.59	0.44	0.036*	0.21–0.95

Abbreviations: CI, confidence interval; HR, hazard ration; LUAD, lung adenocarcinoma.

* *P* < 0.05.

## 4 DISCUSSION

Previous studies revealed several important characteristics of Siglec‐15.^12^, ^16^, ^21^ For one thing, Siglec‐15 is up‐regulated in tumor cells and macrophage, rather than normal tissues, implying the restricted activity within tissue microenvironment (TME). For another, Siglec‐15 shows dramatic immunosuppression on T cell response and Siglec‐15 inhibition reverses T cell inhibition, suggesting Siglec‐15‐specific antibody may restore tumor immunity and inhibit tumor growth. In addition, the expression of Siglec‐15 pathway is independent of the PD‐L1/PD‐1 pathway, indicating that targeting Siglec‐15 might be selected as an alternative therapeutic choice for those barely respond to anti‐PD‐L1/PD‐1 therapy.^22^, ^23^, ^24^

Bioinformatics analyses were firstly performed to investigate a number of expression features of Siglec‐15 in human cancers. For tissue samples, HPA, GEPIA, and TCGA databases all demonstrated the high Siglec‐15 expression in LC. For cell samples, HPA database described the detailed information of Siglec‐15 expression, including in single cell level that Siglec‐15 expression mainly located in macrophages. Moreover, Kmplot database disclosed various prognostic function of Siglec‐15, for both pan‐cancer and LC scenarios. Particularly, high Siglec‐15 expression also indicated favorable treatment outcome when utilizing ICIs (anti‐PD‐1 or anti‐PD‐L1).

A number of previous researches also highlighted the significant attributes and potential of Siglec‐15 in LC. Huang et al. reported that Siglec‐15 positive macrophages (PD‐L1‐independent) facilitated the development of an immunosuppressive TME in LUAD without metastasis, which might be the accomplice element of tumor relapse.^25^ Li et al. stated that patients with low CD8A expression/CD8^+^ T cells infiltration and high Siglec‐15 expression were related to the activation of immunosuppressive signature and metabolism‐related pathway, along with increased infiltration of TAMs.^23^ As for underlying mechanism, Zhang et al. revealed that obesity could accelerate immune evasion of non‐small cell lung carcinoma via TFEB‐dependent up‐regulation of Siglec‐15 and glycolytic reprogramming.^26^

In this present study, we collected tissue samples to perform qPCR, western blotting, and IHC analyses to further investigate Siglec‐15 expression in LUAD. The qPCR test with 16 LUAD samples showed substantially elevated expression of Siglec‐15 in cancer tissues than that in noncancerous tissues. Western blotting analysis with three LUAD samples validated that the protein level of Siglec‐15 was also up‐regulated in cancer tissues. Then a LUAD cohort containing 93 cases in a TMA was prepared, and the result of IHC analysis verified the expression characteristics of Siglec‐15. Analogously, Shafi et al. reported the increased Siglec‐15 expression in both tumor and immune cells in four types of cancer (lung, breast, head, and neck squamous cell carcinoma and bladder cancer)^15^; Quirino et al. stated high Siglec‐15 expression could be observed in neoplastic tissues in gastric cancer.^27^ Furthermore, high Siglec‐15 protein expression statistically associated with TNM stage, and the data also consistent with the previous studies that showed the carcinogenic roles of Siglec‐15 in human cancer, for instance participating the development and progression of retroperitoneal liposarcoma,^28^ promoting immune evasion of acute lymphoblastic leukemia,^29^ and facilitating cancer cell migration of hepatoma.^30^

In survival analysis, univariate analysis screened several important parameters that dramatically correlated with overall survival of 93 LUAD patients, such as Siglec‐15 expression, N status, and TNM stage. Although Siglec‐15 was failed to be identified as an independent prognostic factor for LUAD prognosis, Kaplan–Meier curve also depicted that LUAD patients with elevated Siglec‐15 expression encountered a crucial disappointing outcome than that of patients with low expression. The results of survival statistics were consistent with a study reported by Jiang et al., which summarized the unfavorable OS implication of Siglec‐15 in solid tumors by performing a meta‐analysis.^31^

Interestingly, several studies reported the diverse prognostic characteristics of Siglec‐15. Hao et al. revealed that Siglec‐15 expression was not associated with the prognosis of early NSCLC.^21^ Jiang et al. concluded that Siglec‐15 expression demonstrated a dramatically worse OS but favorable DSS simultaneously.^31^ In our previous study, a novel Siglec‐15 antibody was prepared and showed encouraging tumor‐inhibitory effectiveness in LUAD by modulating macrophage polarization, suggesting a detrimental factor of Siglec‐15 role in cancer management.^32^ Nevertheless, Zhou et al. illustrated that Siglec‐15 was associated with a better pathological response and more favorable survival in ESCC patients receiving neoadjuvant chemoradiotherapy, implying a beneficial element of Siglec‐15 role in the treatment of human cancer.^33^ The reason of these inconsistent or even conflicting data may be due to the multifunctional qualities of Siglec‐15. As a immunosuppressive molecule, Siglec‐15 in different expression site or various tumor type could give rise to multifarious activities in TME.

Moreover, there are several issues in this present research. For one thing, we did not enroll LUAD cell lines to detect Siglec‐15 expression, nor did we perform a serial of knockdown or rescue experiments. For another, the mechanism of Siglec‐15 function is not fully investigated, and it remains barely known about how Siglec‐15 modulates cellular–cellular communication or signaling pathway in LUAD TME. Further and thorough researches that enroll larger cancer types, explore the cellular crosstalk, and elucidate the potential mechanisms of Siglec‐15 performance are of great significance to prove and deepen our current results.

## 5 CONCLUSION

In all, up‐regulation of Siglec‐15 expression was observed in LUAD and elevated Siglec‐15 expression correlated with TNM stage. High expression of Siglec‐15 implied unfavorable overall survival in LUAD patients. Siglec‐15 might be identified as a novel prognostic biomarker in LUAD, and targeting Siglec‐15 may provide a promising strategy for LUAD immunotherapy.

## AUTHOR CONTRIBUTIONS

Lin Wang and Yuan Mao designed the study. Haijun Sun, Qilong Du, and Yuyu Xu collected the tissue samples. Haijun Sun and Yuyu Xu performed the PCR and WB experiments. Li Xu and Junrong Yang performed the IHC analysis. Haijun Sun, Qilong Du, and Cheng Rao performed the statistics. Haijun Sun and Qilong Du drafted the manuscript. Lin Wang and Yuan Mao supervised the study. All authors had full access to the data in the study and take responsibility for the integrity of the data and the accuracy of the data analysis.

## CONFLICT OF INTEREST STATEMENT

The authors declare no potential conflicts of interest with respect to the research, authorship, and publication of this article.

## ETHICS STATEMENT

Written informed consent was obtained from the patients for the publication of this study and the use of any accompanying images. The study protocol was approved by the Ethics Committee of The Fourth Affiliated Hospital of Nanjing Medical University (20230303‐k098).

## Supporting information


**Figure S1.** Siglec‐15 protein concentrations in the pan‐cancer cohort (https://www.proteinatlas.org/ENSG00000197046-SIGLEC15/disease).

**Figure S2.** Siglec‐15 expression suggests poor prognosis in Pan‐cancer circumstance (P = 0.0299, https://kmplot.com/analysis).

## Data Availability

The datasets used and analyzed during the current study are available from the corresponding author on reasonable request.
