# Supplementary figures and images for: The expression characteristic and prognostic role of Siglec‐15 in lung adenocarcinoma
Source: Clin Respir J. 2024 May 9;18(5):e13772. doi: 10.1111/crj.13772 (PMC11082535; doi:10.1111/crj.13772)

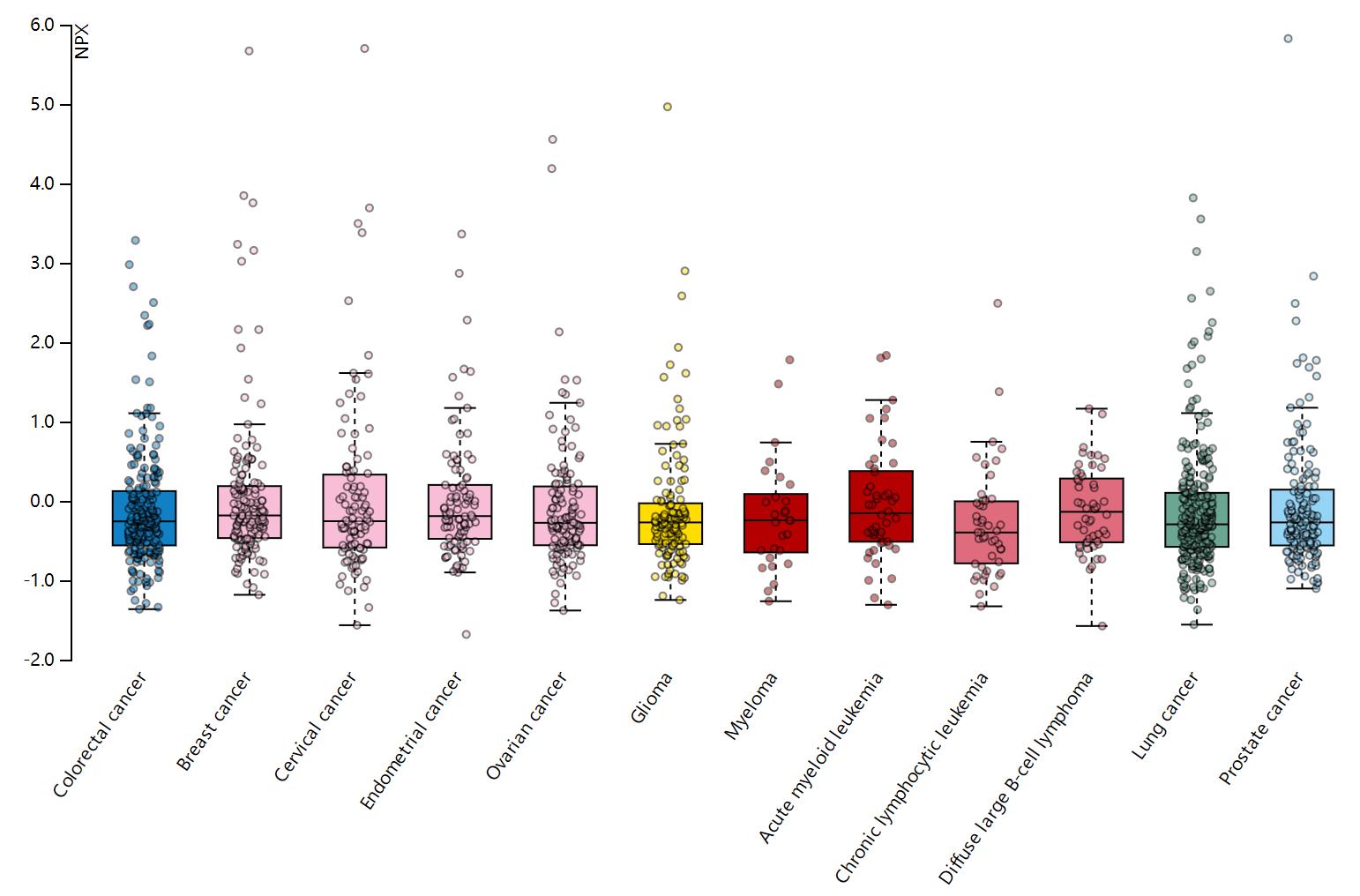

Supplement: Supplementary file 1 — Figure S1. Siglec‐15 protein concentrations in the pan‐cancer cohort (https://www.proteinatlas.org/ENSG00000197046-SIGLEC15/disease). [file CRJ-18-e13772-s001.jpg]

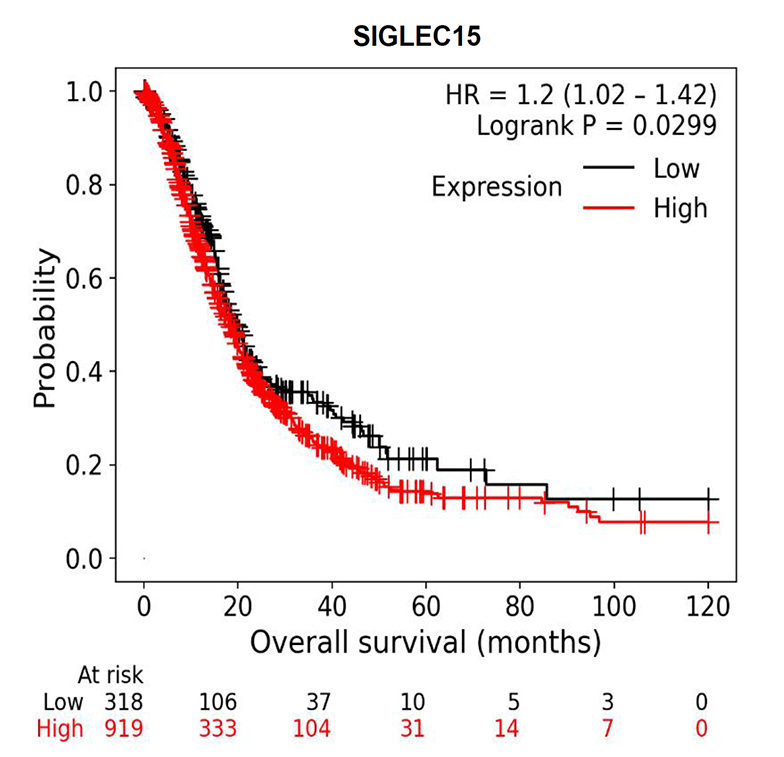

Supplement: Supplementary file 2 — Figure S2. Siglec‐15 expression suggests poor prognosis in Pan‐cancer circumstance (P = 0.0299, https://kmplot.com/analysis). [file CRJ-18-e13772-s002.jpg]
